# Effect of Greenhouse Gases Dissolved in Seawater

**DOI:** 10.3390/ijms17010045

**Published:** 2015-12-30

**Authors:** Shigeki Matsunaga

**Affiliations:** National Institute of Technology, Nagaoka College, Nishikatakai 888, Nagaoka 940-8532, Japan; matsu@nagaoka-ct.ac.jp; Tel./Fax: +81-258-34-9252

**Keywords:** molecular dynamics, carbon dioxide, methane, hydrogen carbonate, seawater, structure, transport properties, thermal properties

## Abstract

A molecular dynamics simulation has been performed on the greenhouse gases carbon dioxide and methane dissolved in a sodium chloride aqueous solution, as a simple model of seawater. A carbon dioxide molecule is also treated as a hydrogen carbonate ion. The structure, coordination number, diffusion coefficient, shear viscosity, specific heat, and thermal conductivity of the solutions have been discussed. The anomalous behaviors of these properties, especially the negative pressure dependence of thermal conductivity, have been observed in the higher-pressure region.

## 1. Introduction

The reason for the global warming of the earth’s climate seems undoubtedly attributable to the increasing of greenhouse gases, especially carbon dioxide (CO_2_) in the atmosphere. The consumption of fossil fuels since the industrial revolution has released a large amount of CO_2_ into the air, the concentration of which is expected to exceed 400 ppm in 2015 [1]. Therefore, technologies for reducing CO_2_ emissions are a matter of the utmost importance, and have widely been investigated, including CO_2_ absorbing processes into liquids [2]. In addition to the soluble property of CO_2_ in water, the increasing concentration of CO_2_ in the atmosphere and the warmer climate may accelerate the dissolution of CO_2_ into seawater, which can simply be modeled as a sodium chloride (NaCl) aqueous solution [3].

However, few molecular dynamics (MD) studies on CO_2_ and NaCl aqueous solutions are available in the literature [4]. Although many experimental studies on CO_2_ dissolved into a NaCl aqueous solution have been published, the solubility of CO_2_ and/or the stability of the solution are their main purpose [4,5,6]. To the best of our knowledge, the thermal conductivity of the CO_2_ absorbed NaCl aqueous solution by MD simulation has not been reported up to the present. Considering the situation, we wish to show the results of a MD simulation of CO_2_ absorbed into a NaCl aqueous solution. The CO_2_ concentration is postulated to be that of saturation. The pressure of the solution in the MD calculation corresponds to the depth of the ocean [7].

The dissolved CO_2_ molecules, however, react with water molecules to create bicarbonate (HCO_3_^−^) and carbonate (CO_3_^2−^) ions in the seawater. The concentrations of these ions, or which ions are more abundant, depend on the acidity of the seawater. The concentration of CO_3_^2−^ ions increases in alkaline regions, whereas CO_2_ molecules are dominant in acidic regions. Because the average pH of seawater is ordinarily between 7.9 and 9.0, the more abundant ion is HCO_3_^−^, *i.e.*, the following reaction is postulated [3]:

CO_2_ + H_2_O → HCO_3_^−^ + H^+^ (1)

which has the function of keeping the pH of seawater constant to a certain extent, and guarantees the charge neutrality of the whole system. As the continuous investigation, we will show the results of MD on seawater saturated with HCO_3_^−^ ions as a more realistic model [8].

Although methane is another the greenhouse gas, the methane and water system has attracted more attention as an energy resource, *i.e.*, the methane hydrate, which is formed in the low temperature and high pressure region at the bottom of the sea [9]. The dissolution of the methane into seawater and into the atmosphere also seems to affect the thermal environment of the earth. Many MD studies have been reported on the methane and water system; however, the nucleation or the structural properties in the mixture with pure water are their main purpose [10,11]. In this study, we wish to show the results of a MD simulation of the methane and NaCl aqueous solution system, as a model of methane dissolved into seawater. The change of structure, the coordination number, and the thermal conductivity will be discussed under the various pressures, corresponding to different sea depths [12]. We believe the results of these investigations will be a part of the fundamental data of the ocean environment.

## 2. Results and Discussion

In this section, first the results of MD simulation are described for the CO_2_ and NaCl aqueous solution system; then for the system that includes water molecules, Na^+^, Cl^−^, H^+^, and HCO_3_^−^ ions; and finally for the methane and NaCl aqueous solution system.

### 2.1. The CO_2_ and NaCl Aqueous Solution

**Figure 1:**
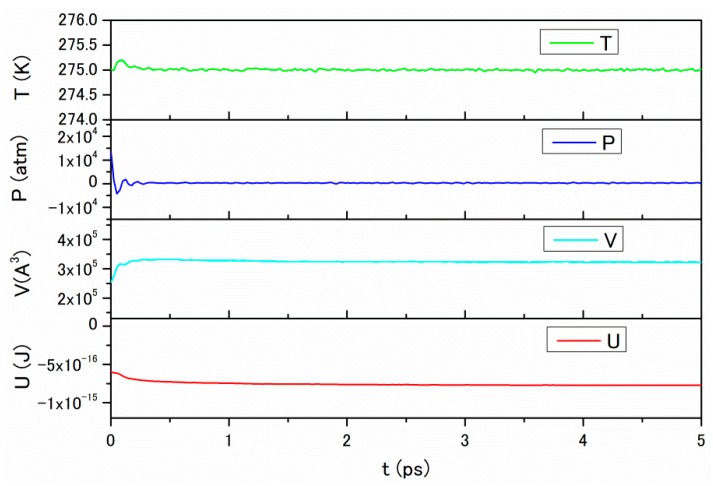
The monitor parameters of the system during 50,000 steps under 30 MP at 275 K. T: temperature, P: pressure, V: volume, U: internal energy.

In the study for CO_2_ and NaCl aqueous solution, the equilibrium MD (EQMD) is used to obtain the structure, the coordination number, the mean square displacement, and the specific heat. The fundamental procedure of EQMD is same as that in our previous works dealing with molten salts [13,14]. As mentioned in the previous section, the thermal conductivity is evaluated using the non-equilibrium MD (NEMD) [15,16]. The rigid body models are used for liquid water using the transferable potential functions of 4-site model (TIP4P) [17] and for CO_2_ molecules. The MD calculation is executed in the number of constituent molecules, the temperature, and the pressure (NTP) constant condition [18,19,20]. The applied pressure varies from 0.5 to 100 MP, or $100\times 10^{6}\;P$, which is equivalent to an ocean depth of 40 to 10,000 m. MD is carried out for 50,000 steps with 0.1 fs time interval. The concentration of NaCl in the water is the same as that of seawater, or 1.1 mol % NaCl. The concentration of CO_2_ is adjusted to that of a saturated solution. The used molecular numbers for the structure calculation at 275 K are listed in Table 1. The parameters of the system are monitored for 50,000 steps under 30 MP at 275 K to guarantee convergence (Figure 1). About 5000 molecules (15,000 atoms) are used for the thermal conductivity calculation so as to contribute to rapid convergence.

**Table 1:** The number CO_2_ and NaCl molecules used in the aqueous solution at 275 K.

Pressure	Water	Na^+^	Cl^−^	CO_2_
0.5 MP (283 K)	10,000	112	112	48
30 MP	10,000	112	112	575
60 MP	10,000	112	112	750
100 MP	10,000	112	112	812

**Figure 2:**
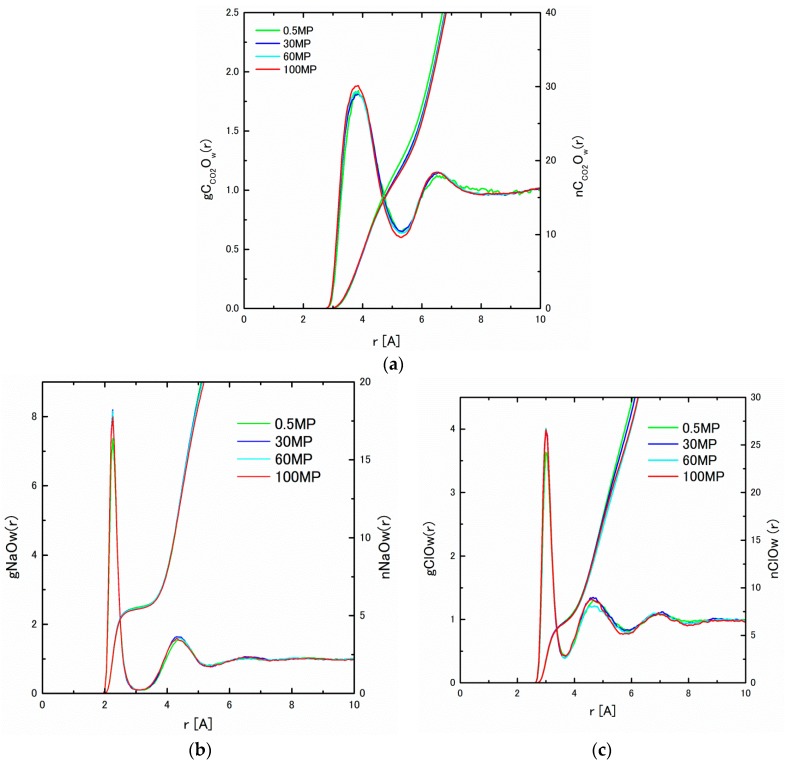
(**a**) gC_CO2_O_w_(*r*) and nC_CO2_O_w_(*r*) under 0.5 (283 K), 30, 60, and 100 MP at 275 K; (**b**) gNaO_w_(*r*) and nNaO_w_(*r*); and (**c**) gClO_w_(*r*) and nClO_w_(*r*) under 0.5 (283 K), 30, 60, and 100 MP at 275 K.

The pair distribution functions, g*_ij_*(*r*), and the integrated coordination number, n*_ij_*(*r*), have been obtained from the MD results, and are shown in Figure 2a–c under 0.5, 30, 60 and 100 MP. The slight enhancement of the first peak heights of g*_ij_*(*r*) of CO_2_–H_2_O, Na^+^–H_2_O, Cl^−^–H_2_O and H_2_O–H_2_O can be observed under higher pressures, which may correspond to the cage formation by H_2_O molecules. To examine the structural change by pressure more clearly, we have calculated the coordination number, or the value of n*_ij_*(*r*) at the first minimum of g*_ij_*(*r*). The obtained coordination numbers are listed in Table 2. As a matter of fact, the pressure dependence of the coordination numbers, nNaO_w_(*r*) and nClO_w_(*r*), are in the margin of error, although a slight decreasing tendency can be observed. The decreasing coordination number for nC_CO2_O_w_(*r*) is obviously seen. These facts might be evidence of the cage formation around the CO_2_ molecules, because the cage structure yields the space around CO_2_ molecules, thereby decreasing the coordination numbers.

**Table 2:** The coordination numbers for nC_CO2_O_w_(*r*), nNaO_w_(*r*), and nClO_w_(*r*).

Pressure	nC_CO2_O_w_(*r*)	nNaO_w_(*r*)	nClO_w_(*r*)
0.5 MP	20.1	5.5 ± 0.1	6.4 ± 0.2
30 MP	19.3		
60 MP	19.1		
100 MP	18.7		

**Figure 3:**
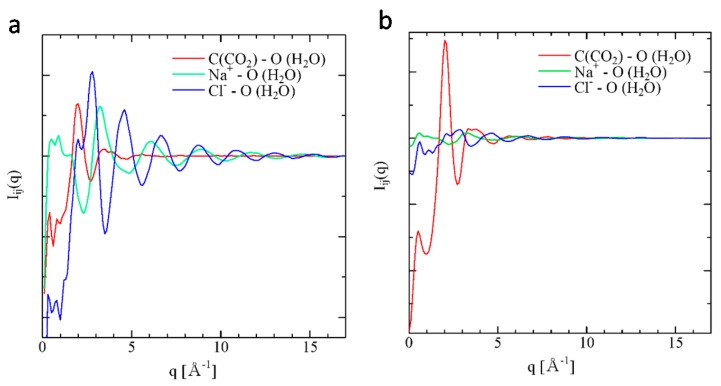
(**a**) I*_ij_*(*r*) at 283 K, 0.5 MP and (**b**) I*_ij_*(*r*) at 275 K, 100 MP.

The interference functions, I*_ij_*(*q*), which is also obtainable from the neutron diffraction experiment, can be calculated from the Fourier transformation of the pair distribution function g*_ij_*(*r*) as expressed in Equation (10) [21]. The evaluated I*_ij_*(*q*)s from g*_ij_*(*r*)s obtained by MD are shown in Figure 3a,b. A sharp peak of I_C-Ow_(*q*) at 2 [Å^−1^] at 283 K, 0.5 MP in Figure 3a is extremely enhanced at 275 K, 100 MP in Figure 3b. Although the peak heights of I_Na-Ow_(*q*) and I_Cl-Ow_(*q*) are similar to that of I_C-Ow_(*q*) at 283 K, 0.5 MP in Figure 3a, their heights decrease and their widths are broadened at 275 K, 100 MP in Figure 3b. These structure changes may also be attributed to the structure formation of water molecules, as they are enhanced as the concentration of CO_2_ increases.

**Figure 4:**
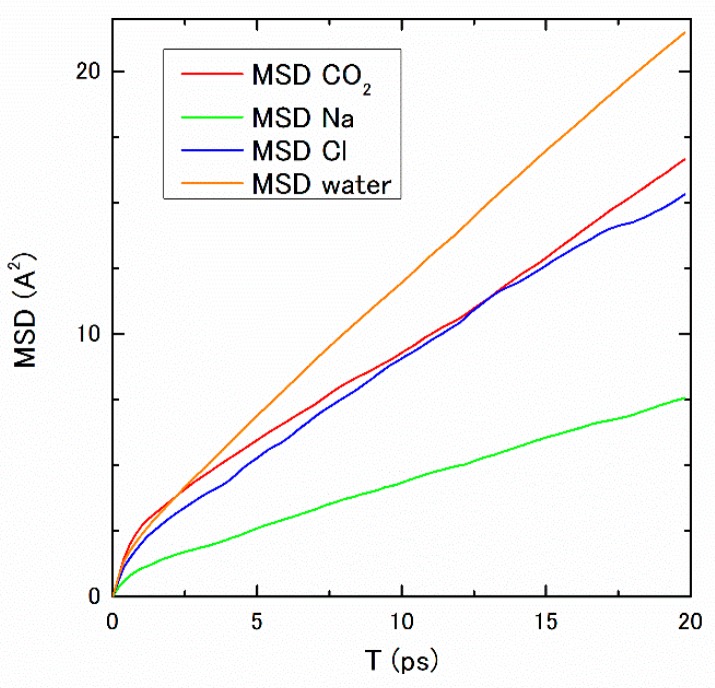
The MSDs of constituent molecules and ions under pressure 30 MP and temperature 275 K.

To examine the pressure dependence of the transport properties, the diffusion coefficients of constituent molecules and ions are calculated, which are obtainable from the inclination of the mean square displacement (MSD) using Equation (11). The obtained MSD for 30 MP is shown in Figure 4. Although small oscillations still remain in the diffusion coefficient for Cl^−^, the inclinations of MSDs seem to converge until $5\times 10^{-12}s$ (5 ps) and the slopes are kept for longer periods, to a certain extent. The pressure dependence of the diffusion coefficients, D*_i_*, is listed in Table 3. The decreasing tendency of the diffusion coefficients also suggests the structure formation in the higher-pressure regions.

**Table 3:** The pressure dependence of D*_i_* (×10^−5^ cm^2^/s) at 275 K.

Pressure	CO_2_	Na^+^	Cl^−^	Water
0.5 MP	1.42	1.28	1.58	2.65
30 MP	1.24	0.70	1.13	1.65
60 MP	1.17	0.55	0.94	1.47
100 MP	0.91	0.52	0.70	1.30

Next, we calculate the thermal conductivity to investigate the thermal properties of 1.1 mol % NaCl aqueous solution with saturated CO_2_. To the best of the author’s knowledge, this is the first report on the thermal conductivity of CO_2_ and NaCl aqueous solution system obtained by MD. The perturbation is applied to the system in the thermal equilibrium at time *t* = 0. According to the Green-Kubo (G-K) formula, the thermal conductivity λ is obtained using Equations (14)–(16).

**Figure 5:**
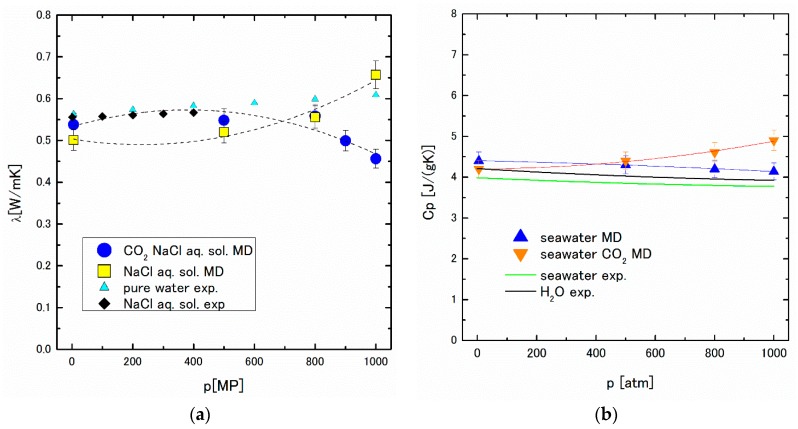
(**a**) The pressure dependence of the thermal conductivity; (**b**) The pressure dependence of the specific heat at the constant pressure. In (**a**,**b**), the horizontal axes show pressure. The abbreviates “aq.”, “sol.”, “exp.” stand for “aqueous”, “solution”, and “experiment”, respectively. The dotted lines in (**a**) and the orange and blue lines in (**b**) are drawn to guide the reader’s eyes.

The thermal conductivity of the aqueous solution of the molecule containing a few atoms can also be derived by NEMD, postulating that the contribution from the atom in the same molecule is omitted from the perturbation current [22]. The thermal conductivity obtained by NEMD under various pressures is shown in Figure 5a, alongside the experimental data of pure water and 1 mol % NaCl aqueous solution [23,24]. The results of NEMD for the saturated concentration of CO_2_ in 1.1 mol % NaCl aqueous solution deserves special mention: the thermal conductivity decreases above 80 MP, which forms a striking contrast with the positive pressure dependence of other thermal conductivity data on solutions, in which CO_2_ is not included. This anomaly of thermal conductivity also signifies the structure change of the CO_2_ absorbed NaCl aqueous solution under high pressure.

Furthermore, the specific heat at constant pressure, C*_p_*, obtained by MD using Equation (13) is shown in Figure 5b. The significant increase of C*_p_* under higher pressure can be seen, although the experimental and MD data for pure water and seawater show a decreasing tendency of C*_p_* against pressure. These results suggest the possibility of heat storage in the depths of the sea.

### 2.2. The System Including Water Molecule, Na^+^, Cl^−^, H^+^, and HCO_3_^−^ Ions

As stated in the previous subsection, the thermodynamic properties of CO_2_ and NaCl aqueous solution show the anomalous features under high pressure. These facts prompt us to a further study. As mentioned in the previous section, the CO_2_ molecule is ionized to form HCO_3_^−^ in the neutral pH. In this study, we wish to show the results of MD on seawater saturated with HCO_3_^−^ ions as a more realistic model. MD is performed in 1.1 mol % NaCl aqueous solution with saturated HCO_3_^−^ from 0.44 to 7.97 mol %, corresponding to 5–1200 atm [25]. Then, for the calculation, MD cell contains 2500 TIP4P, 28 Na^+^, 28 Cl^−^, and 11 to 219 H^+^ and HCO_3_^−^.

**Figure 6:**
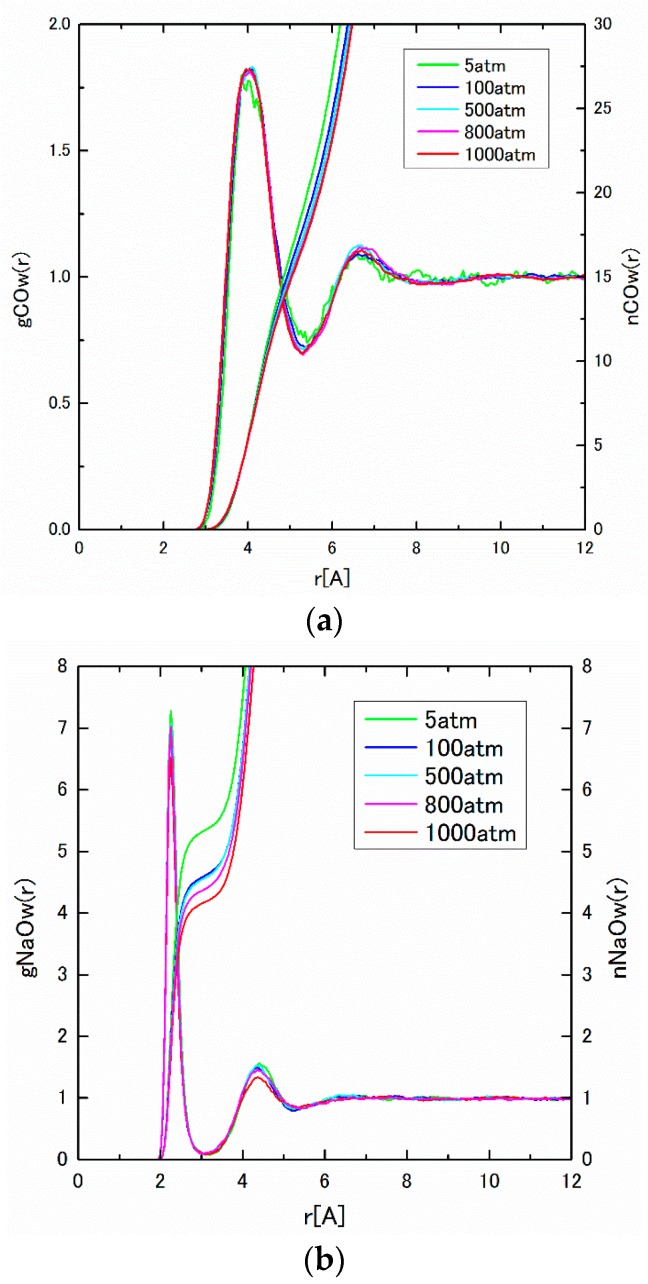
(**a**) g_COw_(*r*) and n_COw_(*r*) under pressures of 5–1000 atm; and (**b**) g_NaOw_(*r*) and n_NaOw_(*r*) under pressures of 5–1000 atm.

**Figure 7:**
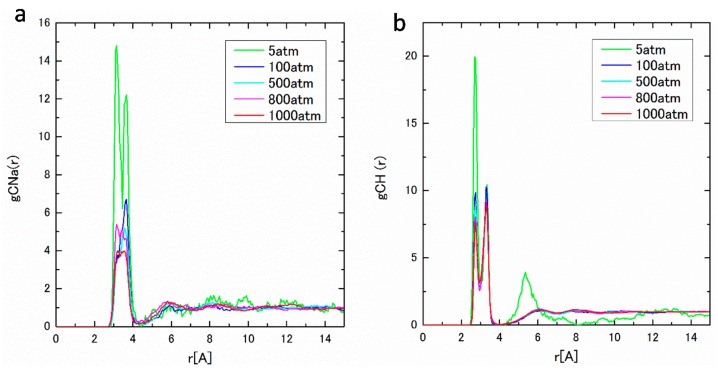
(**a**) g_CNa_(*r*) under pressures of 5–1000 atm; and (**b**) g_CH_(*r*) under pressures of 5–1000 atm.

The pair distribution function, g*_ij_*(*r*), and the integrated coordination number, n*_ij_*(*r*), obtained from MD results are shown in Figure 6 and Figure 7 under pressures from 5 to 1000 atm. As seen in Figure 6a, g_COw_(*r*) between C(HCO_3_^−^) and O(water) has a pronounced peak at around 4 Å. The coordination number of water around a HCO_3_^−^ is estimated to be 17, which agrees well with those in the literature [26]. The sharp first peaks of g_NaOw_(*r*) are found around 2.3 Å in Figure 6b, which shows the close distance between cations and water molecules. Figure 6a,b correspond to those of C(CO_2_)–O(water) and Na–O(water) in Figure 2a,b. To confirm the pressure dependence of the coordination number, which was seen in the Section 2.1, we calculate the coordination number to the first minimum of g*_ij_*(*r*). The obtained results are listed in Table 4. The negative pressure dependence of the coordination number is also observed, which is also the collaborating evidence of the structure formation around HCO_3_^−^ ions. In Figure 7a,b, g_CNa_(*r*), C(HCO_3_^−^)–Na^+^, g_CH_(*r*), and C(HCO_3_^−^)–H^+^ have pronounced two split peaks from 2.5 to 4.0 Å, which may be attributed to the asymmetric form of HCO_3_^−^.

**Table 4:** The coordination numbers for n_COw_(*r*), n_NaOw_(*r*), and n_ClOw_(*r*).

Pressure	n_COw_(*r*)	n_NaOw_(*r*)	n_ClOw_(*r*)
5 atm	19.1	5.31	6.55
100 atm	18.3	4.65	6.49
500 atm	17.4	4.57	6.00
800 atm	17.3	4.43	5.51
1000 atm	16.5	4.18	5.21

**Figure 8:**
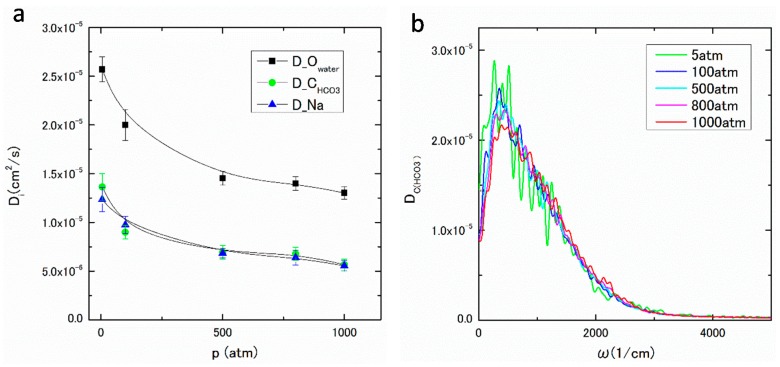
(**a**) D*_i_* under pressures of 5–1000 atm; and (**b**) $D_{C\left({\text{HCO}}_{3}^{-}\right)}\left(\omega \right)\;$under pressures of 5–1000 atm.

In Figure 8a, the diffusion coefficients of water molecule, HCO_3_^−^ and Na^+^, or D_O(water)_, D_C(HCO3-)_, and D_Na_, obtained from MSD defined by Equation (11), are plotted against pressure. The obtained values agree well those in the literature [27]. As seen in Figure 8a, all D*_i_* s decrease as the pressure increases. It is noteworthy that D_C(HCO3-)_, and D_Na_ show similar pressure dependence. These features of g*_ij_*(*r*) s and D*_i_* s suggest that the complex [HCO_3_·(H_2_O)_n_]^−^ is expected to be formed in the solution. Then, the clusters {Na^+^·[HCO_3_·(H_2_O)_n_]^−^} and {H^+^·[HCO_3_·(H_2_O)_n_]^−^} should be compounded to hold the local charge neutrality. Similar structures have also been found in the aqueous solutions. According to the *ab initio* MD study of Na^+^ in aqueous solution, the n-coordinate hydration structures, such as Na(H_2_O)_n_^+^, have been found [28]. In an aqueous solution of CaCO_3_, Ca(HCO_3_)_2_(H_2_O)_4_ and Ca(HCO_3_)_3_(H_2_O)_2_^−^ are predicted to be stable [29].

The frequency dependent diffusion coefficient, $D_{i}\left(\omega \right)$, can be derived from the velocity auto-correlation function (VAF), or $\langle v_{i}\left(t\right)\cdot v_{i}\left(0\right)\rangle$ using Equation (12). The obtained $D_{C\left({\text{HCO}}_{3}^{-}\right)}\left(\omega \right)$ for HCO_3_^−^ under various pressures are observed in the THz or the infrared region as shown in Figure 8b. The peak of $D_{C\left({\text{HCO}}_{3}^{-}\right)}\left(\omega \right)$ around 300 1/cm under low pressure is very close to the frequency of water caused by the translational cage effect. The peak position slightly sifts to the higher frequency around 500 1/cm, and the small hump around 1000 1/cm can be observed, which are comparable to the CO_2_–H_2_O intermolecular vibrational frequency [30].

**Figure 9:**
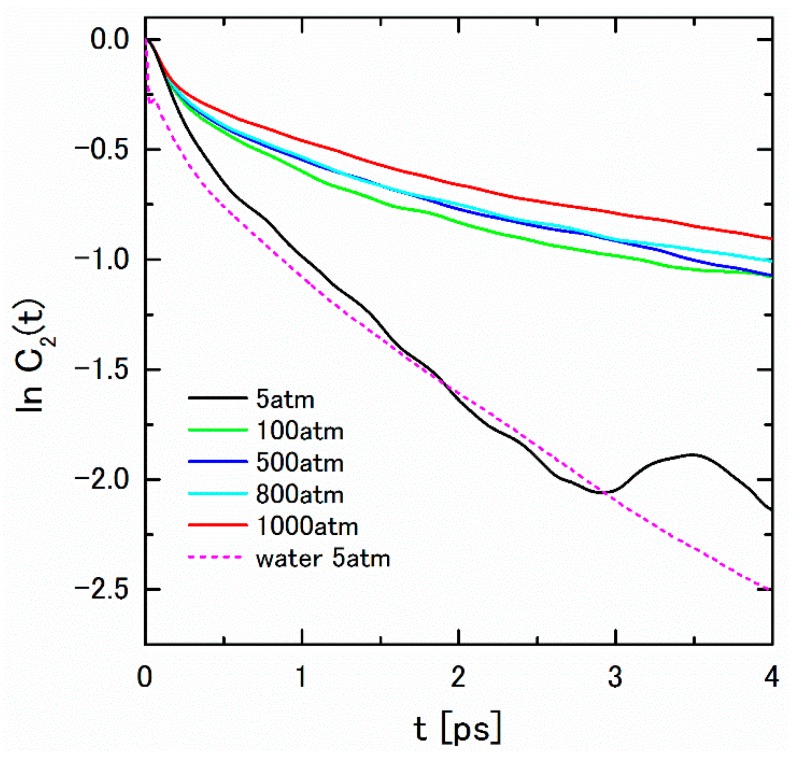
ln*C*_2_(*t*) of HCO_3_^−^ under pressures of 5–1000 atm with log*C*_2_(*t*) of water under 5 atm.

In order to evaluate the lifetime of the complex [HCO_3_·(H_2_O)_n_]^−^, we calculate the rotational correlation function, *C*_2_(*t*), of HCO_3_^−^ ion defined by Equation (19). The *C*_2_(*t*) is thought to be affected by the relaxation of the interaction between HCO_3_^−^ and the surrounding water molecules. The logarithm plot of the obtained *C*_2_(*t*)s in various pressure are shown in Figure 9. The lifetime can be evaluated from the inclination of the linear part of ln*C*_2_(*t*). The graph of ln*C*_2_(*t*) of HCO_3_^−^ at 5 atm is extremely similar to that of water molecule. The oscillatory behavior at 3–4 ps may be interpreted as the “free-rotor” motion, which is observed in the dilute phase [15]. The estimated lifetime at 5 atm is 1.6 ps; on the other hand, those of at 100–1000 atm is 6.7 ± 1.3 ps, which is comparable to the relaxation time of H-bond in the aqueous carbonate solution [31]. The slight positive pressure dependence of the relaxation time also suggests the structure formation in the higher-pressure region.

**Figure 10:**
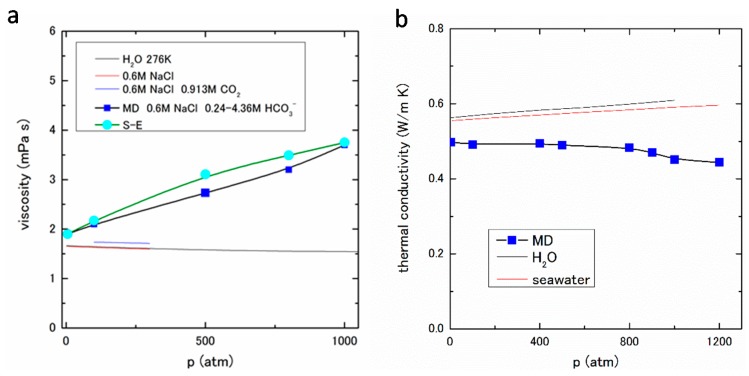
(**a**) Viscosity under pressures of 5–1000 atm; and (**b**) Thermal conductivity under pressures of 5–1200 atm.

For the next stage, according to the G-K formula, the shear viscosity is calculated using Equation (17). The shear viscosities obtained by MD are shown in Figure 10a with the experimental values for pure water, 0.6 M NaCl aqueous solution, and 0.6 M NaCl and 0.913 M CO_2_ aqueous solution in the literature [32,33]. The pressure dependence of the present result (0.6 M NaCl with saturated HCO_3_^−^) is positive, whereas those of experimental values are negative. This fact suggests that the interaction between HCO_3_^−^ and water, and/or other constituents increases as the pressure increases. A significant increasing tendency of viscosity with increasing mole fraction of dissolved CO_2_ has also been observed in the viscosity measurement of CO_2_ saturated seawater at 303 to 333 K under constant pressure 10 to 20 MPa [34]. To ensure the MD result, the shear viscosity is also estimated from the diffusion coefficient obtained by MD using the Stokes–Einstein (S-E) relation for a spherical particle, which is expressed as

(2) $$D=\frac{k_{B}T}{\xi }=\frac{k_{B}T}{6\pi \eta r}$$

where the parameters ξ and η stand for the friction constant and the shear viscosity, respectively. If the shear viscosity η is determined at a certain CO_2_ concentration *c*_0_, then η(*c*) at any concentration *c* could be estimated using the following equation [35]:

(3) $$\frac{D\left(c\right)}{D\left(c_{0}\right)}=\frac{\eta \left(c_{0}\right)}{\eta \left(c\right)}$$

which is known as the Walden’s rule. The calculated η(*c*), from Equation (3), is also plotted in Figure 10a, which agrees with the MD results to a certain extent.

As mentioned in the previous subsection, we have calculated the thermal conductivity of 1.1 mol % NaCl aqueous solution with saturated CO_2_ by NEMD method [7,16]. In this study, we adopt the same method using the saturated HCO_3_^−^ ion in 1.1 mol % NaCl aqueous solution. As will be described in Section 3, the thermal conductivity λ is expressed as Equations (14)–(16). The obtained results of the thermal conductivity by NEMD are shown in Figure 10b. The experimental data of pure water and seawater are also shown in Figure 10b [23,36]. The negative pressure dependence of thermal conductivity is clearly seen in the MD result; on the other hand, those of the experimental data are positive.

As stated already, some anomalous results have been obtained by MD in the transport and thermal properties of 1.1 mol % NaCl aqueous solution saturated with HCO_3_^−^. The experimental thermal conductivity data of electrolyte aqueous solutions show positive pressure dependence, and *negative* concentration dependence of electrolyte [37]. These phenomena have been explained to some extent by the extension of the additivity of the thermal conductivity by considering the interaction between components [37,38]. The results in this study might be influenced by the above-mentioned contradictory effects to the thermal conductivity, pressure and concentration. In addition, the results are also supposed to be attributed to the complex and/or the cluster formation in the solution.

### 2.3. The Methane and NaCl Aqueous Solution

Next, we will show the MD result of the methane and NaCl aqueous solution. The fundamental procedure of MD is the same as described in the previous subsections. The water (TIP4P) and the methane are treated as rigid body molecules. The concentration of NaCl is adjusted to be the same as that of seawater, 1.1 mol %. The number of CH_4_ in the MD cell is determined using the solubility data of CH_4_ in seawater [39]. The numbers of particles used in MD are listed in the Table 5.

**Table 5:** The number of CH_4_ and NaCl molecules used in the aqueous solution at 275 K.

Pressure	Water	Na^+^	Cl^−^	CH_4_
10 MP	10,000	112	112	26
30 MP	10,000	112	112	46
60 MP	10,000	112	112	63
100 MP	10,000	112	112	82

**Figure 11:**
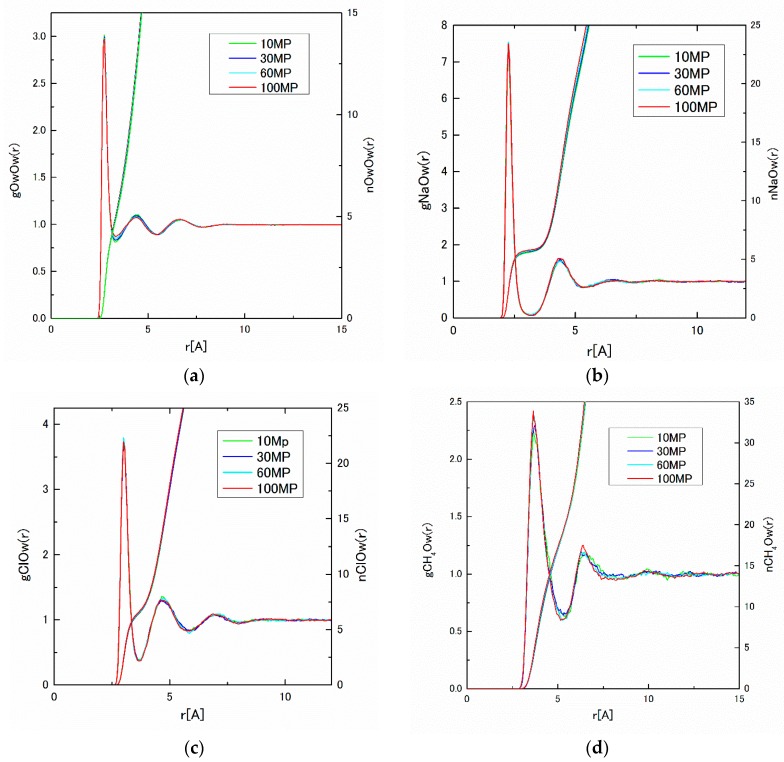
(**a**) g_OwOw_(*r*) and n_OwOw_(*r*); (**b**) g_NaOw_(*r*) and n_NaOw_(*r*); (**c**) g_ClOw_(*r*) and n_ClOw_(*r*) ; and (**d**) g_CH4Ow_(*r*) and n_CH4Ow_(*r*) under pressures of 10–100 MP.

From the MD results, the pair distribution functions, g*_ij_*(*r*)s, and the integrated coordination numbers, n*_ij_*(*r*)s, have been obtained for 10 to 100 MP, which are shown in Figure 11a–d. Although the pressure dependence of g*_ij_*(*r*) is not large, the slight change of the first peak height for CH_4_–CH_4_, and the depth for the first minimum for H_2_O–H_2_O can be observed, which may correspond to the cage formation in the solution. The water coordination number, n*_ij_*(*r*), of the first hydration shell around the solute is calculated to the first minimum of g*_ij_*(*r*) using Equation (9). The obtained water coordination numbers calculated under pressures of 10–100 MP are listed in Table 6. The slight decreasing tendency of water molecules around CH_4_ has been detected, which might also be attributed to the cluster formation around CH_4_ molecules.

**Table 6:** Water coordination number under pressures of 10–100 MP.

Pressure	Water	Na^+^	Cl^−^	CH_4_
10 MP	4.56	5.50	6.57	20.58
30 MP	4.56	5.53	6.58	20.25
60 MP	4.56	5.69	6.58	19.95
100 MP	4.48	5.77	6.58	19.65

**Figure 12:**
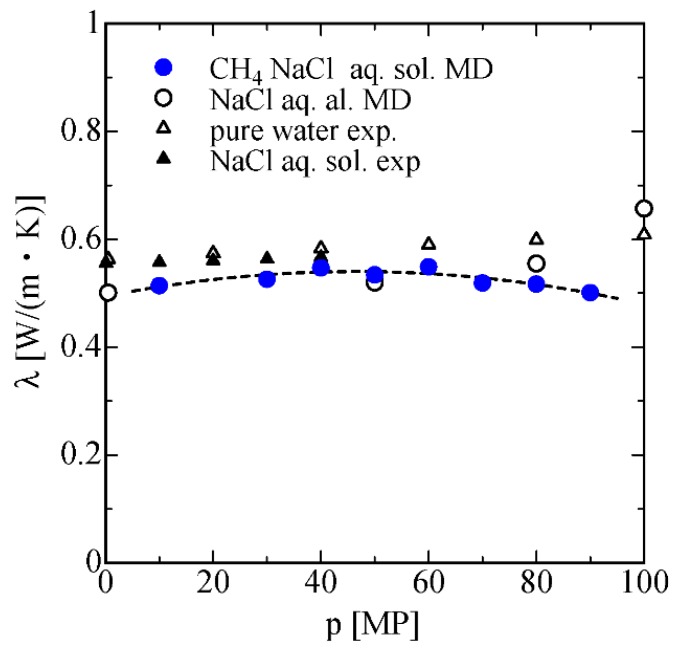
The pressure dependence of the thermal conductivity of CH_4_ and NaCl aqueous solution, and NaCl aqueous obtained by molecular dynamics (MD), alongside the experimental data.

Finally, the pressure dependence of thermal conductivity of methane and NaCl aqueous solution is obtained by NEMD using Equations (14)–(16). The obtained results are shown in Figure 12 alongside the experimental data and MD data for NaCl aqueous solution and pure water. The negative pressure dependence of thermal conductivity in higher pressure is also observed. This result might be attributed to the structure change or the clathrate formation around the CH_4_ molecule in the high-pressure region, which is consistent with the discussion regarding the decreasing of coordination number in the solution.

## 3. Procedure

The essential numerical procedure of this MD simulation study is the same as our previous works on aqueous solutions [7,8,12,40]; however, the fundamental part of the procedure is described as follows for the reader’s benefit.

The water is treated as the rigid body model, TIP4P [17]. The potential function for TIP4P is expressed in the charged Lennard-Jones (L-J) type potentials as

(4) $$\phi_{ij}\left(r\right)=\frac{z_{i}z_{j}e^{2}}{r}+4\varepsilon \left\{{\left(\frac{\sigma }{r}\right)}^{12}+{\left(\frac{\sigma }{r}\right)}^{6}\right\}$$

In the equations hereafter, *i* and *j* stand for the constituent atoms; *z_i_* is the charge for the constituent species *i*; and *e* is the elementary charge. The used parameters are listed in Table 7.

**Table 7:** The potential parameters for TIP4P.

The Atom Pair	*z_i_*	*z_j_*	ε (gA^2^/fs^2^)	σ (A)
O–O	0	0	1.0769 × 10^−28^	3.153650
O–XX	0	−1.04	0	0
O–H	0	0.52	0	0
XX–XX	−1.04	−1.04	0	0
XX–H	−1.04	0.52	0	0
H–H	0.52	0.52	0	0

The interactions between Na^+^ and Cl^−^, TIP4P-Na^+^, and TIP4P-Cl^−^ are expressed as [41],

(5) $$\phi_{ij}\left(r\right)=\frac{z_{i}z_{j}e^{2}}{r}+\frac{C}{r^{9}}-\frac{D}{r^{6}}$$

The used parameters are listed in Table 8.

**Table 8:** The potential parameters between Na^+^, Cl^−^ and TIP4P water.

The Atom Pair	*z_i_*	*z_j_*	*C* (10^−19^ JA^9^)	*D* (10^−19^ JA^6^)
Na^+^–Na^+^	1.0	1.0	1.5565 × 10^2^	8.3931
Cl^−^–Cl^−^	−1.0	−1.0	2.4808 × 10^4^	2.6162 × 10^2^
Na^+^–Cl^−^	1.0	−1.0	3.2015 × 10^3^	4.6860 × 10
Na^+^–O	1.0	0	5.8553 × 10^2^	2.3463 × 10
Na^+^–H	1.0	0.52	0	0
Na^+^–XX	1.0	−1.04	0	0
Cl^−^–O	−1.0	0	9.2132 × 10^3^	1.3099 × 10^2^
Cl^−^–H	−1.0	0.52	0	0
Cl^−^–XX	−1.0	−1.04	0	0

The interactions between other solute ions and water molecules are also expressed in the charged L-J type potentials [42,43] as

(6) $$\phi_{ij}\left(r\right)=\frac{z_{i}z_{j}e^{2}}{r}+\frac{A}{r^{12}}-\frac{B}{r^{6}}$$

The parameters of the interactions between CO_2_ molecule, HCO_3_^−^ ion, Na^+^, Cl^−^ and water molecule, taken from the literature, are listed in Table 9, Table 10, Table 11 and Table 12.

**Table 9:** The potential parameters between CO_2_–CO_2_, CO_2_–Na^+^, CO_2_–Cl^−^, and CO_2_–TIP4P water.

The Molecule Pair	The Atom Pair	*z_i_*	*z_j_*	*A* (kcal A^12^/mol)	*B* (kcal A^6^/mol)
CO_2_–CO_2_	C 2–C 2	0.4578	0.4578	3.2481 × 10^6^	1.1680 × 10^3^
	C 2–O 2	0.4578	−0.2289	1.1110 × 10^6^	8.1233 × 10^2^
	O 2–O 2	−0.2289	−0.2289	3.8000 × 10^5^	5.6498 × 10^2^
CO_2_–Na^+^	C 2–Na^+^	0.4578	1.0000	7.9212 × 10^5^	8.3121 × 10^2^
	O 2–Na^+^	−0.2289	1.0000	3.3217 × 10^5^	5.3912 × 10^2^
CO_2_–Cl^−^	C 2–Cl^−^	0.4578	−1.0000	3.4465 × 10^6^	1.5642 × 10^3^
	O 2–Cl^−^	−0.2289	−1.0000	1.1789 × 10^6^	1.0879 × 10^3^
CO2–TIP4P	C 2–O 1	0.4578	0.0000	1.0834 × 10^6^	7.6092 × 10^2^
	C 2–XX	0.4578	−1.04	0	0
	C 2–H	0.4578	0.5200	1.9677 × 10^5^	2.3881 × 10^2^
	O 2–O 1	−0.2289	0.0000	3.7057 × 10^5^	5.2922 × 10^2^
	O 2–XX	−0.2289	−1.04	0	0
	O 2–H	−0.2289	0.5200	6.7305 × 10^4^	1.6609 × 10^2^

**Table 10:** The potential parameters between H^+^–H^+^, HCO_3_^−^–HCO_3_^−^, TIP4P–H^+^, TIP4P–HCO_3_^−^, Na^+^–H^+^, and Na^+^–HCO_3_^−^.

The Molecule Pair	The Atom Pair	*z_i_*	*z_j_*	*A* (kcal A^12^/mol)	*B* (kcal A^6^/mol)
H^+^–H^+^	H^+^–H^+^	1.0000	1.0000	1.7199 × 10^4^	3.2337 × 10^1^
HCO_3_^−^–HCO_3_^−^	C 2–C 2	1.2149	1.2149	1.1713 × 10^6^	6.6752 × 10^2^
	C 2–O 2	1.2149	−0.8727	5.3596 × 10^5^	4.5224 × 10^2^
	C 2–O 1	1.2149	−0.9424	5.3596 × 10^5^	4.5224 × 10^2^
	C 2–H	1.2149	0.4194	1.5060 × 10^5^	1.5134 × 10^2^
	O 2–O 2	−0.8727	−0.8727	2.3212 × 10^5^	2.9808 × 10^2^
	O 2–O 1	−0.8727	−0.9424	2.3212 × 10^5^	2.9808 × 10^2^
	O 2–H	−0.8727	0.4194	6.3567 × 10^4^	9.8477 × 10^1^
	O 1–O 1	−0.9424	−0.9424	2.3212 × 10^5^	2.9808 × 10^2^
	O 1–H	−0.9424	0.4194	6.3567 × 10^4^	9.8477 × 10^1^
	H–H	0.4194	0.4194	1.7199 × 10^4^	3.2337 × 10^1^
TIP4P–H^+^	O 1–H^+^	0	1.0000	6.3567 × 10^4^	9.8477 × 10^1^
	XX–H^+^	−1.0400	1.0000	0	0
	H–H^+^	0.5200	1.0000	1.7199 × 10^4^	3.2337 × 10^1^
TIP4P–HCO_3_^−^	O 1–C 2	0	1.2149	5.3596 × 10^5^	4.5224 × 10^2^
	O 1–O 2	0	−0.8727	2.3212 × 10^5^	2.9808 × 10^2^
	O 1–O 1	0	−0.9424	2.3212 × 10^5^	2.9808 × 10^2^
	O 1–H	0	0.4194	6.3567 × 10^4^	9.8477 × 10^1^
	XX–C 2	−1.0400	1.2149	0	0
	XX–O 2	−1.0400	−0.8727	0	0
	XX–O 1	−1.0400	−0.9424	0	0
	XX–H	−1.0400	0.4194	0	0
	H–C 2	0.5200	1.2149	1.5060 × 10^5^	1.5134 × 10^2^
	H–O 2	0.5200	−0.8727	6.3567 × 10^4^	9.8477 × 10^1^
	H–O 1	0.5200	−0.9424	6.3567 × 10^4^	9.8477 × 10^1^
	H–H	0.5200	0.4194	1.7199 × 10^4^	3.2337 × 10^1^
Na^+^–H^+^	Na^+^–H^+^	1.0000	1.0000	8.9597 × 10^4^	1.7676 × 10^2^
Na^+^–HCO_3_^−^	Na^+^–C 2	1.0000	1.2149	7.9212 × 10^5^	8.3121 × 10^2^
	Na^+^–O 2	1.0000	−0.8727	3.3217 × 10^5^	5.3912 × 10^2^
	Na^+^–O 1	1.0000	−0.9424	3.3217 × 10^5^	5.3912 × 10^2^
	Na^+^–H	1.0000	0.4194	8.9597 × 10^4^	1.7676 × 10^2^

**Table 11:** The potential parameters between Cl^−^–H^+^, Cl^−^–HCO_3_^−^, and H^+^–HCO_3_^−^.

The Molecule Pair	The Atom Pair	*z_i_*	*z_j_*	*A* (kcal A^12^/mol)	*B* (kcal A^6^/mol)
Cl^−^–H^+^	Cl^−^–H^+^	−1.0000	1.0000	2.0880×10^5^	3.1983 × 10^2^
Cl^−^–HCO_3_^−^	Cl^−^–C 2	−1.0000	1.2149	3.4465 × 10^6^	1.5642 × 10^3^
	Cl^−^–O 2	−1.0000	−0.8727	1.1789 × 10^6^	1.0879 × 10^3^
	Cl^−^–O 1	−1.0000	−0.9424	1.1497 × 10^6^	1.0191 × 10^3^
	Cl^−^–H	−1.0000	0.4194	2.0880 × 10^5^	3.1983 × 10^2^
H^+^–HCO_3_^−^	H^+^–C 2	1.0000	1.2149	1.9677 × 10^5^	2.3881 × 10^2^
	H^+^–O 2	1.0000	−0.8727	6.7305 × 10^4^	1.6609 × 10^2^
	H^+^–O 1	1.0000	−0.9424	6.5635 × 10^4^	1.5558 × 10^2^
	H^+^–H	1.0000	0.4194	1.1921 × 10^4^	4.8828 × 10

**Table 12:** The potential parameters between CH_4_–CH_4_, TIP4P–CH_4_, Na^+^–CH_4_, and Cl^−^–CH_4_.

The Molecule Pair	The Atom Pair	*z_i_*	*z_j_*	*A* (kcal A^12^/mol)	*B* (kcal A^6^/mol)
CH_4_–CH_4_	C 1–C 1	−0.3744	−0.3744	3.2481 × 10^6^	1.1680 × 10^3^
	C 1–H	−0.3744	0.0936	1.9677 × 10^5^	2.3881 × 10^2^
	H–H	0.0936	0.0936	1.1921 × 10^4^	4.8828 × 10^1^
CH_4_–TIP4P	C 1–O 1	−0.3744	0	1.0834 × 10^6^	7.6092 × 10^2^
	H–O 1	0.0936	0	6.5635 × 10^4^	1.5558 × 10^2^
	C 1–XX	−0.3744	−1.0400	0	0
	H–XX	0.0936	−1.0400	0	0
	C 1–H	−0.3744	0.5200	1.9677 × 10^5^	2.3881 × 10^2^
	H–H	0.0936	0.5200	1.1921 × 10^4^	4.8828 × 10^1^
CH_4_–Na^+^	C 1–Na^+^	−0.3744	1.0000	7.9212 × 10^5^	8.3121 × 10^2^
	H–Na^+^	0.0936	1.0000	8.9598 × 10^4^	1.7676 × 10^2^
CH_4_–Cl^−^	C 1–Cl^−^	−0.3744	−1.0000	3.4465 × 10^6^	1.5642 × 10^3^
	H–Cl^−^	0.0936	−1.0000	2.0880 × 10^5^	3.1983 × 10^2^

The solute ions and molecules and water molecules are at first randomly placed at the lattice points, which are obtained by dividing each side by the number “*n*” that satisfies the following relation,

(*n* − 1)^3^ < (the total number of water molecules and solute ions and molecules) < *n*^3^ (7)

Then, the relaxation of the first configuration using the Monte Carlo method is executed by the following procedure:

(a) One ion or molecule is randomly selected.
(b) One degree of freedom is selected randomly for the set of {ξ} = {r, Ω, θ}, where “r” is the translation of the center of mass, “Ω” is the rotation around the center of mass, and “θ” is the rotation around the bond axis of the molecule.
(c) The selected degree of freedom ξ of the selected ion or molecule is changed by Δξ, then the now set {ξ}′ is created. The increment of freedom “Δr” is randomly determined from 0 to Δ*r*_max_. A random degree is applied for “ΔΩ” and “Δθ”.
(d) The above procedures (a)–(c) are repeated for the defined number of steps until the ensemble average is calculated.
(e) The potential energy difference Δϕ between the previous configuration {ξ} and the new configuration {ξ}′ is calculated.
(f) The decision whether the new configuration {ξ}′ is adopted or not is made according to the following condition:
  If Δϕ < 0, then the new configuration {ξ}′ is adopted,
  If Δϕ > 0, then a uniform random number η is compared to the Boltzmann factor exp(-Δϕ /kT),
  If η ≤ exp(-Δϕ/kT), then {ξ}′ is adopted as the new configuration,
  If η > exp(-Δϕ/kT), then {ξ}′ is not adopted.

The (a)–(f) procedures above are repeated to obtain the lower energy configuration, which we then adopt as the first configuration of the MD calculation.

The cut of distance for the van der Waals interaction is 15 Å. The Ewald method is used for the calculation of the Coulomb interaction, in which the square of the cut off distance in the reciprocal lattice space is 27. The static properties of the solutions are calculated in the NTP constant condition for 100,000–500,000 steps with 0.1 fs being one time step [18,19,20]. The very short time step is adopted so as to detect the fast movement of H^+^ and water molecules.

The pair distribution function, g*_ij_*(*r*), can be firstly obtained from a time series data of coordinates of ions as [15]

(8) $$g_{ij}\left(r\right)=\left(V/N_{i}N_{j}\right)\overset{N_{i}}{\underset{k}{\sum }}c_{kj}\left(r-\frac{\Delta r}{2},\;r+\frac{\Delta r}{2}\right)/\left(4\pi r^{2}\Delta r\right)$$

where *N_i_* and *N_j_* are the numbers of the ion species *i* and *j*, respectively. *V* is the volume of the cell. *c_ik_* is the number of ion *k* in the spherical shell of the thickness of Δ*r* at the distance *r* from the ion *i*. The distance dependent coordination number, or the integrated coordination number, n*_ij_*(*r*), is defined as

(9) $$n_{ij}\left(r\right)=\underset{n<r/\Delta r}{\sum }(N_{j}/V)g_{ij}\left(n\cdot \Delta r\right)$$

The interference functions for neutron I*_ij_*(*q*) are obtained from g*_ij_*(*r*), which is expressed as [21]

(10) $$I_{ij}\left(q\right)=c_{i}b_{i}c_{j}b_{j}/{\left(\underset{k}{\sum }c_{k}b_{k}\right)}^{2}\int_{0}^{\infty }4\pi r^{2}\rho_{0}\left(g_{ij}\left(r\right)-1\right)\frac{\sin\left(rq\right)}{qr}dr$$

where *c*_i_ is the atomic fraction of the *i*-type atoms; *ρ*_0_ is the average number density; and *b*_i_ is the neutron scattering amplitude of the *i*-type atom.

The diffusion coefficient for *i*-type atom, D*_i_*, can be obtained from MSD, which is defined as

(11) $$D_{i}=\underset{t\to \infty }{\lim}\frac{1}{6t}\langle {\left|r_{i}\left(t\right)-r_{i}\left(0\right)\right|}^{2}\rangle$$

VAF is calculated to examine the dynamical and transport properties, which is expressed as $\langle v_{i}\left(t\right)\cdot v_{i}\left(0\right)\rangle$. The frequency dependent diffusion coefficient, $D_{i}\left(\omega \right)$, can be obtained from VAF as

(12) $$D_{i}\left(\omega \right)=\frac{1}{3}\int_{0}^{\infty }\langle v_{i}\left(t\right)\cdot v_{i}\left(0\right)\rangle \text{cos}\omega t\;dt$$

where ω *=* 2π *f*; *f* is the frequency, and D*_i_*(ω = 0) = D*_i_*.

The thermodynamic properties are also important for the study of solutions. The specific heat at the constant pressure is expressed as

(13) $$C_{p}=\frac{1}{M}\frac{dH}{dT}=\frac{1}{M}\left(\frac{dU}{dT}+P\frac{dV}{dT}\right){|}_{p}$$

In order to calculate the thermal conductivity, NEMD method is used. In the NEMD method, the average of energy flux overtime is performed to avoid the margin of error caused by the integration of the energy flux autocorrelation function, which is used in the direct method or the EQ method [16]. The system reaches thermal equilibrium by EQMD, then the perturbation is applied at time *t* = 0. The thermal conductivity is expressed as the G-K formula [15],

(14) $$\lambda =\frac{1}{Vk_{B}T^{2}}\int_{0}^{\infty }\langle J_{x}\left(\tau \right)J_{x}\left(0\right)\rangle d\tau$$

In Equation (14), *J_x_*(τ) stands for the *x* component of the perturbation current; *V* the volume of the system; *k_B_* the Boltzmann constant; and *T* the temperature of the system. The applied perturbation *F*_ext_ is related to the average of the perturbation current <*J_x_*(τ)>*_t_*,

(15) $${\langle J_{x}\rangle }_{t}=\frac{F_{ext}}{k_{B}T}\int_{0}^{t}\langle J_{x}\left(\tau \right)J_{x}\left(0\right)\rangle d\tau$$

Then, the relation between the thermal conductivity and the perturbation is expressed as [15]

(16) $$\lambda =\frac{1}{VF_{ext}T}\underset{t\to \infty }{\lim}{\langle J_{x}\rangle }_{t}$$

According to the G-K formula, the shear viscosity is expressed by the integration of the autocorrelation function of an off-diagonal element of the stress tensor in the long wave length limit *k* → 0 [15],

(17) $$\eta =\frac{1}{kTV}\int_{0}^{\infty }\underset{k\to 0}{\lim}\langle \sigma_{k}^{xz}\left(t\right)\sigma_{-k}^{xz}\left(0\right)\rangle$$

The reorientation of linear molecules is expressed as the time-correlation function defined as [15]

(18) $$C_{l}\left(t\right)=\langle P_{l}\left[u_{i}\left(t\right)\cdot u_{i}\right]\rangle$$

where ***u****_i_*(t) is unit vector parallel to the principal axis of the molecule *i*. *P*_l_(x) is the Legendre polynomial. The rotational correlation function is the case of *l* = 2, which is expressed as

(19) $$C_{2}\left(t\right)=P_{2}\left(\cos\omega t\right)=\frac{1}{2}\langle 3{\left(\cos\omega t\right)}^{2}-1\rangle =\frac{1}{2}\langle 3{\left\{u_{i}\left(t\right)\cdot u_{i}\right\}}^{2}-1\rangle$$

In the case that the decay in polarization anisotropy *C*_2_(*t*) is modeled in the standard exponential form *C*_2_(*t*) = A exp(−*t*/τ), the relaxation time is obtained by the logarithm plot of *C*_2_(*t*).

The main part of MD is performed using SIGRESS ME package (Fujitsu) [44] at the supercomputing facilities in Kyushu University. The obtained results from MD simulation are summarized in the previous section.

## 4. Conclusions

MD simulations have been performed for the system CO_2_, HCO_3_^−^, and CH_4_ dissolved in 1.1 mol % NaCl aqueous solution as a seawater model. The pressure dependence of structure, coordination number, diffusion coefficient, frequency distribution of ions, shear viscosity, heat capacity, and thermal conductivity have been examined. It is worth noting that the negative pressure dependence of thermal conductivity has been detected in these solutions, which is considered to be attributed to the structure change of the solutions under high pressure.
